# Trying to see, failing to focus: near visual impairment in Down syndrome

**DOI:** 10.1038/srep20444

**Published:** 2016-02-05

**Authors:** Lesley Doyle, Kathryn J. Saunders, Julie-Anne Little

**Affiliations:** 1 School of Biomedical Sciences, University of Ulster, Cromore Road, Coleraine, Northern Ireland, BT52 1SA.

## Abstract

The majority of individuals with Down syndrome (DS) do not exhibit accurate accommodation, with the aetiology of this deficit unknown. This study examines the mechanism underlying hypoaccommodation in DS by simultaneously investigating the ‘near triad’ – accommodation, vergence and pupillary response. An objective photorefraction system measured accommodation, pupil size and gaze position (vergence) under binocular conditions while participants viewed an animated movie at 50, 33, 25 and 20 cm. Participants were aged 6–16 years (DS = 41, controls = 76). Measures were obtained from 59% of participants with DS and 99% of controls. Accommodative response was significantly less in DS (p < 0.001) and greater accommodative deficits were associated with worsening visual acuity (p = 0.02). Vergence responses were as accurate in DS as in controls (p = 0.90). Habitual pupil diameter did not differ between groups (p = 0.24) but reduced significantly with increasing accommodative demand in both participants with and without DS (p < 0.0001). This study is the first to report simultaneous binocular measurement of the near triad in DS demonstrating that hypoaccommodation is linked to poor visual acuity. Vergence responses were accurate indicating that hypoaccommodation cannot be dismissed as a failure to visually engage with near targets, but rather is a consequence of underlying neurological or physiological deficits.

Accommodation (focusing) deficits have been widely reported in individuals with Down syndrome (DS)^1^,^2^,^3^,^4^,^5^,^6^,^7^,^8^ leading to impaired near vision. As DS is the most common cause of intellectual impairment in humans, and children with DS are known to be visual learners, it is important that visual needs are met to maximise educational development and quality of life^9^. The precise aetiology and mechanism of the accommodative deficit remains unidentified. From the current body of research, a number of possible aetiologies have been proposed including; limited cognitive ability or inattention to the target^3^, a mechanical defect similar to that found in presbyopia^2^,^4^,^6^,^8^,^10^, a sensory defect of the accommodative system^4^,^8^ or an abnormal relationship between accommodation and convergence mechanisms^3^,^6^,^10^.

Typically, when a near object is viewed, a simultaneously focused and single retinal image is achieved by the activation and neural control of the accommodative and vergence response systems^11^. Pupil size also plays a role in determining the clarity of the retinal image, with a decrease in pupil size increasing depth-of-focus. The three components of this combined response to a near target are referred to as the ‘near triad’. It is widely accepted that the accommodative and vergence systems respond to a number of different cues including retinal blur, retinal disparity and proximity cues. However, owing to neural connection between the two systems, accommodation accompanies convergence even in the absence of retinal blur (convergence-accommodation), and convergence accompanies accommodation in the absence of the retinal disparity (accommodative-convergence)^12^,^13^.

The majority of studies investigating accommodative function in DS have explored accommodation and near visual impairment through examining the accuracy of individual participant’s focus on near targets, without consideration of the other components of the triad (vergence and pupil size). No previous reports have evaluated accommodation in conjunction with simultaneous vergence and pupil data and it is currently unclear whether vergence movements or pupil characteristics differ in the DS population. To fully appreciate the accommodative deficit in DS and better investigate its underlying mechanisms it is necessary to evaluate all three aspects of the near triad and their interactions. The aim of the present study is to simultaneously assess accommodation, vergence and pupil response during a near vision task in children with DS and compare the findings to those from typically developing children.

## Methods

### Participants

The study protocol was approved by the University of Ulster Research Ethics committee, the Office for Research Ethics Committees Northern Ireland (ORECNI) and the Research Office of the Western Health and Social Care Trust (WHSCT). Participant recruitment and data collection was carried out in accordance with the Declaration of Helsinki. Recruitment of typically developing control participants was carried out through the University of Ulster Optometry paediatric clinic and a number of participants with DS were also recruited through this route. An information pack was either mailed to the parent/guardian of potential participants or given at their routine eye examination. Seventy-six typically developing children and 18 children with DS aged 6–16 years were recruited in this manner. A further 23 children with DS were recruited through DS support groups and a local paediatric ophthalmology clinic (WHSCT), resulting in a total of 41 participants with DS. Informed consent was obtained from the parent/guardian of all participants and either written or verbal informed assent from all participants prior to inclusion in the study.

Sample size was calculated (95% power, 5% significance) based on the data from two studies reporting accommodative function employing photorefraction in young adult participants^14^ and participants with DS^8^. The power calculation indicated a minimum sample size of 10 participants per group would be necessary to elicit significant group differences in accommodative responses. This figure was doubled in anticipation of 50% success rates for participants with DS^8^.

### Preliminary Measures

All participants attended the University of Ulster Vision Science Research Laboratory. A short clinical history was taken from the parent/guardian. Where available, refractive error was ascertained from their clinical record. Where recent (within 12 months) refractive error data were not available, a full eye examination was carried out prior to inclusion in the study. Where participants were familiar with upper case letters and where cognitive ability allowed, best corrected visual acuity (BCVA) was assessed using the Keeler LogMAR Crowded Acuity Test. Where letter matching or naming proved too challenging the LogMAR Crowded Kay Picture test was used. Acuity testing was conducted monocularly at a distance of 3 m, with full refractive correction in place. The cover/uncover test was used to detect the presence of strabismus and the prism cover test to measure magnitude where present. Inter-pupillary distance (IPD) was also measured manually using a frame rule.

### Assessment of the Near Response Using Photorefraction

In a similar manner to a number of previous studies investigating the near response in children, a video-based infrared eccentric photorefraction system was employed^14^,^15^,^16^,^17^,^18^,^19^. For the present study, refractive power in the vertical meridian, pupil size and gaze position were measured using the PowerRefractor III (PR III) at a sampling rate of 50 Hz. The PR III assesses refractive power using an infrared light source projected through the pupil, with the distribution of the reflected luminance profile across the vertical meridian of the pupil used to determine the sign and magnitude of refractive error. Purkinje image tracking and a pupil edge detection algorithm are used to determine gaze position and pupil size respectively.

**Figure 1:**
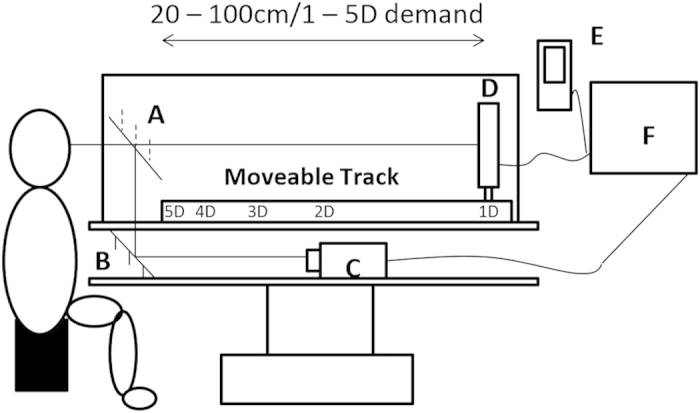
Schematic diagram of the PR III experimental setup. (**A**) IR reflecting mirror, (**B**) Mirror, (**C**) PR camera, (**D**) Moveable target screen, (**E**) Target audio, (**F**) Target control and video monitor.

Figure 1 illustrates the PR III and target configuration used during testing. Participants placed their head on a head rest in front of a viewing aperture. An animated movie target was presented on a LCD monitor, mounted on a moveable longitudinal track and fully enclosed within a matte black casing, ensuring minimal reflections and other distractions. An infrared (IR) reflecting mirror was used to ensure participants were positioned directly in front of the target whilst the camera was masked from view. The target stimulus was a commercially available animated movie containing broadband spatial frequency content in an attempt to capture attention across a wide range of ages and cognitive abilities, to represent a naturalistic and familiar viewing experience and to provide an engaging stimulus for sustained observation. Animated movie targets have been used previously in the study of the near response in infants and children, and have been shown to have a naturalistic amplitude spectra^16^,^17^,^18^. During photorefractive assessment of accommodative function, participants with refractive error ≤−0.50 DS or ≥+0.50 DS and ≤−1.00 DC were corrected either with their habitual spectacles (where appropriate) or using full aperture trial lenses. Trial lenses were placed in a trial frame fitted carefully to minimise variation in back vertex distance and potential reflections.

It has previously been shown that accuracy and variability of photorefraction systems can be improved by individually calibrating measurements for each individual participant^20^,^21^,^22^,^23^. Accordingly, individual refractive power and eye position calibration was attempted on all participants. Calibration was carried out using spherical and prismatic lenses of known power (+4, +3, +2, +1, −2, −4D spheres and 4, 8, 12, 16^Δ^ base in/out prisms) placed in front of one eye in conjunction with an infrared transmitting filter occluding visible light. The refractive error and eye position data collected during this calibration protocol were used to derive a refractive and eye position ‘calibration factor’ that was applied to all subsequent raw data. Where individual calibration data were not available for a participant the group mean calibration factor derived from the appropriate group (DS or control) was applied.

During assessment of accommodative function the target was initially positioned at 1 m (baseline demand) from the participant where it subtended 9.7° by 4.6° of visual angle. The target was moved manually towards the participant to produce accommodative/vergence demands of 1, 2, 3, 4 and 5D/metre angle (MA). Throughout data capture, in an attempt to ensure attention, participants were questioned for engagement with the target. At least five seconds of ‘consistent and good quality’ data were obtained at each demand before the target was moved to another accommodative demand. Data was considered to be ‘consistent and of good quality’ when at least five seconds of uninterrupted data was obtained during which time the participant was fixing on the target. Data capture was interrupted during target displacement to ensure no data were taken during this time. When moved towards the participant, the target was not scaled for proximity. A corresponding video file of the data capture was scrutinised to identify periods of optimal cooperation and data collection for analysis. The video file took the form of a real-time video screen capture of the PR III recording with video feed interruptions used to indicate a change in target position. Video files were also used to reconfirm periods of participant attention to the target and to provide a time stamp for periods of successful and sufficient data capture providing vignettes of data for further analysis. The length of time required for data collection varied dependent on cooperation and the time taken to obtain sufficient data at each demand.

### Data Analysis

Data analyses were performed using Matlab (Mathworks, USA) and Stata statistical software (StataCorp, USA). Raw PR III data files were processed using Matlab including the application of an individual calibration factor, removal of any data captured during blinks, filtering to remove extraneous refractive power data outside of the PR III working range of −7.00 to +5.00 DS, removal of pupil size data outside the PR III working range of 4–8 mm and removal of eye position data outside ±15° 24.

**Figure 2:**
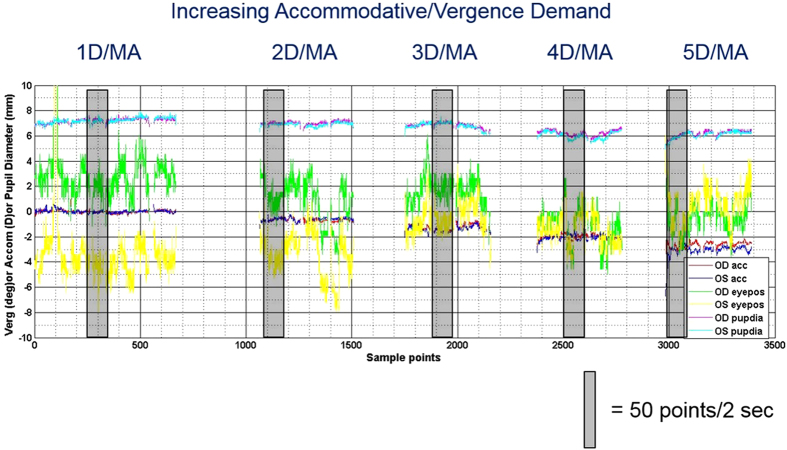
Raw refractive power (accommodative), eye position (vergence) and pupil diameter data for the right and left eyes of an individual participant plotted as a function of time (number of samples with 50 samples taken per second). Stimulus position/accommodative demand is also shown. The grey bars are representative of a two second vignette of data.

Accommodative and vergence data were plotted against time (Fig. 2) and the corresponding video file used to identify and confirm the position and resultant accommodative or vergence demand of the target. A two second vignette of data (approximately 100 samples, incorporating the individual or group calibration factor, where appropriate) were selected for analysis to calculate mean refractive power, gaze position and pupil size at each demand for both right and left eyes. Care was taken to ensure that data chosen for analysis were representative of the response during good cooperation and attention to the target. From gaze position and IPD, vergence in metre angles was calculated. A mean of right and left refractive power and total vergence was plotted against the accommodative or vergence demand and linear regression analysis applied to produce an accommodative and vergence response slope value (i.e. slope of the linear regression line) in accordance with previous studies^16^,^18^,^19^,^25^,^26^. A slope value of one is indicative of an accurate and appropriate change in accommodative or vergence response in line with the change in demand and a value of zero indicates that accommodation or vergence failed to respond to a change in demand. The relationship between the individual components of the near response and visual function (spherical equivalent refraction (SER), visual acuity and ocular posture) were also investigated.

## Results

### Success Rates

Useable photorefraction data were obtained from 24 participants with DS (59%) and 75 control participants (99%). The mean length of recording for control participants and participants with DS was 68 seconds (range: 32–122 seconds) and 123 seconds (range: 57–331 seconds) respectively. Where data were not of useable quality or quantity for further analysis this was due to poor cooperation or intermittent reflection of the infrared light source from spectacle/trial lenses. There was no significant difference in age, SER or BCVA between participants with DS whose data were available for analysis and those whose data were not (p > 0.05). A refractive error calibration factor of sufficient quality for analysis was obtained from 46 control participants (61%) and seven participants with DS (29%), and an eye position calibration value from 56 control participants (75%) and six participants with DS (25%). Where an individual calibration factor was not available the group mean was applied. Group mean calibration factors were 0.99 ± 0.18 and 0.93 ± 0.13 for refractive power calibration and 0.88 ± 0.09 and 0.92 ± 0.12 for eye position calibration in the control and DS groups respectively. There was no significant difference in refractive power or eye position calibration factors between groups (refractive power calibration factor: student’s t-test, t_(51)_ = 0.87, p = 0.39, eye position calibration factor: t_(60)_ = −1.08, p = 0.29).

### Participants

Table 1 summarises the gender, mean age, IPD, SER and BCVA of participants in both the control and DS groups. Strabismus was detected in four participants with DS. Three of four strabismic participants with DS had a manifest deviation during testing, despite full refractive correction. Two of these had manifest angles less than 15^∆^ during testing (one alternating exotropia and one esotropia), thus accommodative responses were averaged from the right and left eyes^26^. In the case of one participant where a larger angle was present, all data from the non-fixing eye was removed during the filtering process as a result of the eye position criterion used (removal of eye position data outside ±15°)^24^. As a result, it was not possible to generate a measure of vergence for this individual and accommodative data were obtained only from the fixing eye to avoid off-axis errors^26^. Participants with DS were significantly younger than control participants (t_(97)_ = 2.68, p = 0.01), significantly more hyperopic (SER OD: t_(96)_ = −1.98, p = 0.03, SER OS: t_(96)_ = −2.27, p = 0.01) and had significantly poorer BCVA (better seeing eye: t_(96)_ = −11.22, p < 0.00001). There was no significant difference in SER between the better and worse seeing eyes in either group (DS: t_(46)_ = 0.23, p = 0.82, Control: t_(148)_ = −0.46, p = 0.78). The SER and VA of the better seeing- or fixing-eye were used for subsequent analyses. At baseline, IPD was significantly larger in control participants in comparison to participants with DS (M-W U test, z_(98)_ = 3.99, p = 0.0001), and the difference remained significant when age was controlled for (p = 0.009).

### Assessment of the Near Response

**Figure 3:**
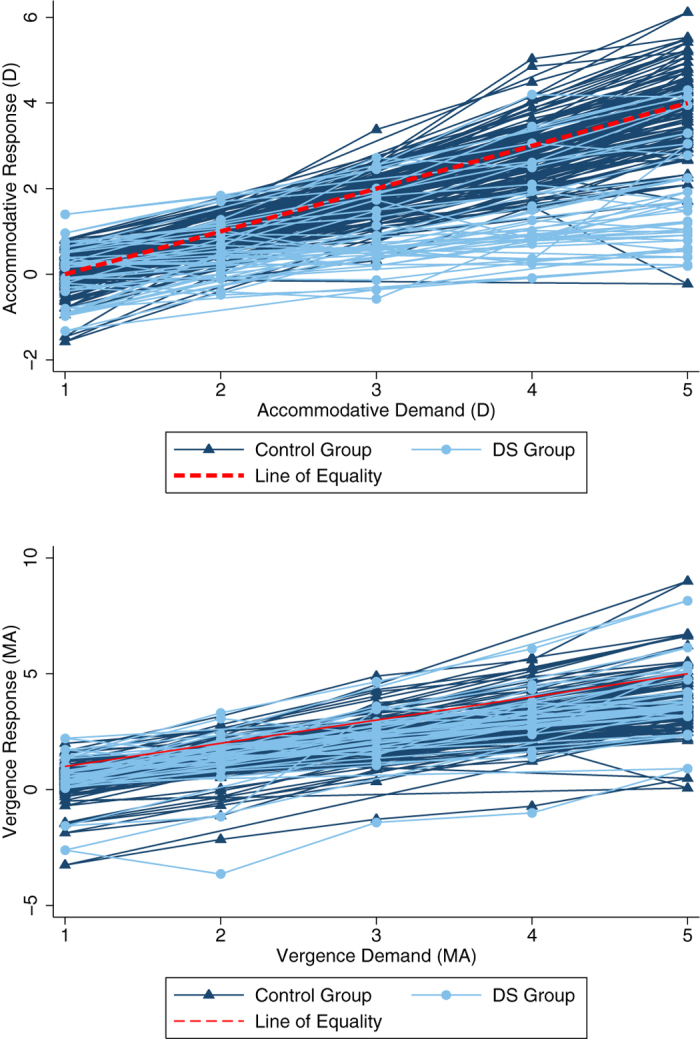
(**a**) Individual accommodative responses to each accommodative demand for all participants by group. Each individual control participant is represented by ▴ and each individual with DS by 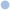. The 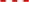 represents an ‘ideal response’ of 1D for each dioptre of accommodative demand from baseline. (**b**). Graph showing the individual vergence responses to each accommodative demand for all participants by group. Each individual control participant is represented by ▴ and each individual with DS by 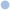. The 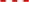 represents an ‘ideal response’ of 1 MA for each metre angle of vergence demand.

**Figure 4:**
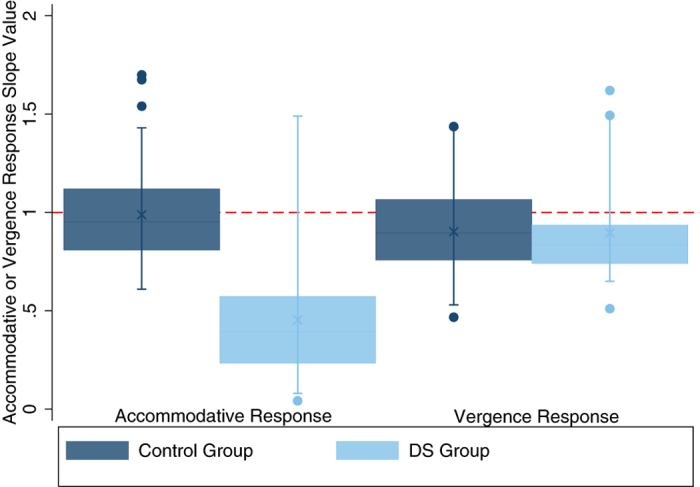
Graph showing the mean, median and range of accommodative and vergence response slope values in both the control and DS groups. The control group are represented by 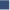 and the DS group by 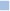. Each box represents the median and interquartile range, the cross represents the mean and the whiskers represent the 5^th^ and 95^th^ centiles. Outliers are represented as single points and the 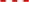 represents the line of equality or an accurate response slope of 1.0.

Figures 3a-3b show the individual accommodative and vergence responses to each accommodative or vergence demand for all participants by group. In order to further analyse accommodative and vergence response data, individual accommodative and vergence slope values were calculated. Table 2 and Fig. 4 summarise the mean (±SD) and range of accommodative and vergence slope values in both groups.

**Figure 5:**
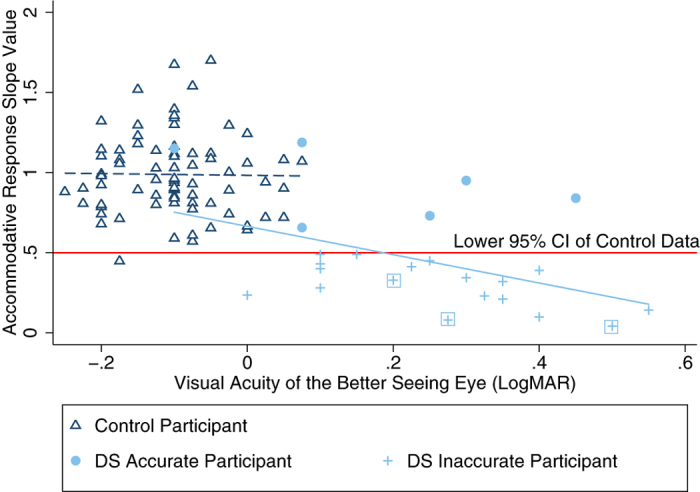
Relationship between accommodative response slope values and BCVA of the better seeing eye in both participants with and without DS. Control participants are represented by Δ and linear regression of these data is represented as 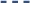 (y = −0.05x + 0.98, Pearson’s r = 0.004, R^2^ = 0.0002, F_(1,73)_ = 0.02, p = 0.90). The lower limit of the 95% confidence interval (CI) of control data is indicated as 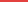. Participants with DS in the DS Accurate subgroup are represented by 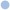 and those in the DS Inaccurate subgroup by 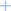. DS participants with manifest strabismus during testing are highlighted with a box surrounding the data point. Linear regression for the DS group as a whole is represented by 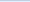 (y = −0.92x + 0.68, Pearson’s r = 0.47, R^2^ = 0.22).

Control participants had significantly larger (more accurate) accommodative response slope values in comparison to participants with DS (two group mean comparison test t_(97)_ = 8.44, p < 0.00001). This difference remained significant when participants with DS and manifest strabismus (n = 3) were removed (t_(96)_ = 7.45, p < 0.00001). Eighteen participants with DS (75%) had accommodative response slope values below the 95% confidence interval (CI)(<0.50) of control participants. Using this criterion, participants with DS were grouped into those whose accommodative responses fell within (DS Accurate) and those whose responses fell outside these limits (DS Inaccurate). All three participants with DS and manifest strabismus during testing had accommodative response slope values outside the 95% CI established from control data. There was no significant difference in BCVA or SER between DS subgroups (BCVA: t_(21)_ = −1.60, p = 0.12, SER: t_(22)_ = −0.35, p = 0.73) however, accommodative response slope value decreased significantly as BCVA worsened in participants with DS as shown in Fig. 5 (linear regression analysis, F_(1,22)_ = 5.77, p = 0.03). The association between accommodative response slope and BCVA in participants with DS remained significant when participants with DS and manifest strabismus (n = 3) were removed from the analysis (p = 0.02).

In contrast to the accommodative response slope, there was no statistically significant difference in vergence response slope values between participants with and without DS (two-group mean comparison test, t_(95)_ = 0.13, p = 0.90). Additionally, there was no significant difference between DS subgroups and controls (Analysis of Variance (ANOVA), F_(1,2)_ = 1.13, p = 0.33). Vergence response slope value did not correlate significantly with any measure of visual function, including BCVA or accommodative response slope value (p > 0.05). Vergence findings remained unchanged when data from participants with DS and manifest strabismus during testing were removed.

Due to associations between accommodative response slope value and refractive error found in previous studies^27^, and the higher magnitude of hyperopia in the DS group of the present study, participants with DS were also compared to a refractive error matched control group (REM control group). Each participant with DS (n = 24) was matched to one or more participants from the control group who had SER within ±1.00 DS of that of the respective participant with DS for both eyes. Table 3 summarises the age, SER and BCVA of the REM Control group (n = 34). Accommodative response slope values remained significantly larger in REM control participants in comparison to participants with DS, with and without the inclusion of participants with DS and manifest strabismus (two-group mean comparison test, t_(55)_ = 5.88, p < 0.00001 and t_(42)_ = 5.18, p < 0.00001, respectively). There was no significant difference in vergence response slope values between participants with DS and REM control participants (two-group mean comparison test, t_(53)_ = 0.70, p = 0.48). This finding remained unchanged when data from participants with DS and manifest strabismus were excluded from analysis (t_(51)_ = 1.04, p = 0.30). Despite a significant difference in IPD between control participants and participants with DS, when considering participants with DS and REM controls, there was no significant difference in IPD between participants with DS and REM controls when age was controlled for (ANCOVA (analysis of covariance), F_(1,1)_ = 1.15, p = 0.29).

In addition to refractive error and eye position, pupil diameter was also sampled binocularly every 20 ms using the PR III. The mean of the pupil diameter samples for the right and left eyes were calculated for each accommodative demand in an identical manner to that used for refractive error and vergence.

**Figure 6:**
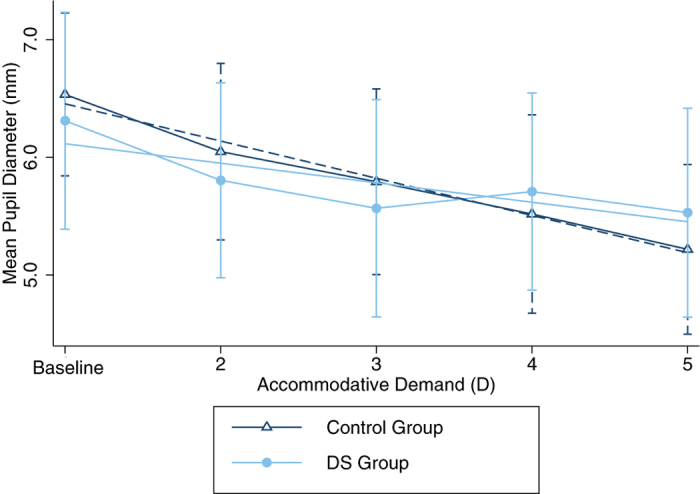
Graph showing mean pupil diameter (±SD) plotted against accommodative demand for participants with and without DS. The control group are represented by 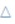 at each demand and 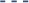 (y = −0.32x + 6.77, Pearson’s r = 0.51, R^2^ = 0.26). The DS group are represented by 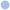 and 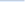 (y = −0.17x + 6.28, Pearson’s r = 0.26, R^2^ = 0.07).

At baseline there was no significant difference in pupil diameter between participants with and without DS (t_(97)_ = 1.26, p = 0.21). Neither did the pupil diameter differ significantly between REM controls and the DS group (t_(55)_ = 0.09, p = 0.93). Figure 6 shows mean pupil diameter of both the control and DS groups at each accommodative or vergence demand. There is a general trend for pupil diameter to decrease with increasing accommodative demand in both groups (control group: repeat measures ANOVA, F_(4,294)_ = 112.69 p <0.0001, DS group: F_(2,92)_ = 12.17, p < 0.0001), with no significant group effect (F_(1,1)_ = 0.21, p = 0.64) or interaction between group and accommodative demand F_(1,4)_ = 2.08, p = 0.08). However, reduction in pupil diameter measured at baseline and at the highest accommodative demand (5D) was significantly greater in the control group (mean change (±SD) = 1.33 ± 0.68 mm) compared to the DS group (mean change (±SD) = 0.78 ± 0.65) two group mean comparison test t_(96)_ = 3.48, p = 0.0004). The change in pupil diameter measured in response to baseline versus a 5D demand did not differ between DS subgroups (DS Accurate mean change = 0.93 ± 0.74, DS Inaccurate subgroup mean change = 0.73 ± 0.65, t_(22)_ = 0.63, p = 0.53).

## Discussion

This study further characterises the accommodative deficit in children with DS previously reported in a number of studies^1^,^2^,^3^,^4^,^5^,^6^,^7^,^8^, with 75% of participants with DS demonstrating impaired accommodation. Importantly, this study describes simultaneous vergence eye movements during accommodative tasks for the first time in a DS group. The data demonstrate that despite hypoaccommodation, participants with DS converge appropriately to targets presented at near in the same manner as typically developing peers, independent of the quality of their accommodative response. Woodhouse *et al*.^3^ argue that whilst cognitive impairments may contribute to poor accommodative performance in DS, they do not fully account for the extent of the deficit found in this population. The data from the present study support this assertion and counter the contention that hypoaccommodation in DS is a consequence of poor attention or compliance associated with the intellectual disability implicit in individuals with DS^3^. If the latter were the case, one would not expect accurate and appropriate vergence responses, which demonstrate deliberate attention to the target stimuli.

The present study demonstrates for the first time an association between accommodative function and visual acuity in children with DS; as BCVA worsens, so does the accuracy of the accommodative response. Previous authors have suggested that reduced visual acuity found in participants with DS may be the cause or consequence of a sustained accommodative lag and subsequent blurred near vision during early development, resulting in subtle bilateral amblyopia^4^. Visual acuity has a long developmental timeframe^28^,^29^, whereas accommodative responses are typically adult-like in the early months of life^30^,^31^,^32^. Impaired accommodation in early life could be detrimental to optimal acuity development, particularly in combination with hyperopia, a common feature in individuals with DS^1^,^4^,^7^,^33^,^34^,^35^. To establish the nature of these interactions in participants with DS, prospective measures of acuity and accommodative function would need to be undertaken from an early age. Using a photorefraction technique, under monocular conditions Anderson *et al*.^8^ did not find a relationship between visual acuity and accommodative function in a group of individuals with DS. However, Anderson *et al*.^8^ employed several different types of acuity measures and their participants spanned a wide range of ages. The narrower age range, availability of binocular accommodation measures and the use of crowded recognition acuity for all participants in the present study provides a clearer picture of the relation between BCVA and accommodative function.

Cregg *et al*.^4^ propose a sensory hypothesis to explain hypoaccommodation in individuals with DS. They contend that the presence of a smaller pupil aperture in individuals with DS results in a greater depth of focus permitting hypoaccommodation to be tolerated, negating the need for accurate accommodation and promoting hypoaccommodation. However, to yield sufficient depth of focus, this theory would require pupil diameters less than 3 mm^18^, much smaller than those observed in the present study. In addition, the lack of significant difference between the DS and control group pupil diameters measured at baseline further contradicts Cregg *et al*.’s^4^ hypothesis as it relates to the pupil aperture’s role in contributing to anomalies in depth of focus in the DS eye. The finding in the present study of similar pupil size in participants with DS and controls does not undermine the notion that the DS eye has a greater tolerance of retinal blur, a possibility suggested by Anderson *et al*.^8^. Anderson *et al*.^8^ reported increased accommodative microfluctuations in participants with DS compared with typically developing control participants and argue that the presence of increased microfluctuations indicates a larger blur threshold in DS thus allowing hypoaccommodation to be tolerated without retinal blur being perceived. Retinal blur and disparity have been shown to be the main cues driving accommodation; with the removal of the retinal disparity and blur cues significantly reducing accommodative responses in the typically developing visual system^19^. It may be hypothesised that if a larger depth of focus is inherent in the DS eye, retinal blur cues may be less effective at driving the accommodative response. Further work is required to ascertain the contribution of individual cues in driving the accommodative and vergence systems in DS.

Our data demonstrate that the vergence response is relatively intact and independent of the accommodative and pupillary response. Despite their close proximity, and mutual innervation by parasympathetic activity of the oculomotor nerve, subsequent to the ciliary ganglion the innervational pathway of the accommodative, pupillary and vergence systems diverge. The inferior division of the oculomotor nerve branches prior to the ciliary ganglion, with the short root, containing fibres deriving from the Edinger-Westphal nucleus, supplying the ciliary muscle and sphincter pupillae muscles only. An abnormality at, or subsequent to the ciliary ganglion may influence initiation of an accommodative and pupillary response without affecting the extraocular muscles used to produce the vergence response. In the present study pupil diameter decreased significantly with increasing accommodative demand in both groups. However, the relationship between change in pupil diameter and increase in accommodative demand was weaker in the DS group in comparison to controls, possibly suggestive of a diminished pupillary response in DS. Further work would be beneficial in a larger sample to fully explore these findings.

Neuromuscular differences in smooth muscle innervation or function, affecting the ciliary muscle may exist in individuals with DS, while the skeletal extraocular muscles function normally. A number of early studies using atropine in individuals with DS reported an increased sensitivity to these agents resulting in a greater degree and duration of dilation, perhaps suggestive of ciliary body and iris plant abnormalities^36^,^37^,^38^,^39^. Sacks and Smith^39^ also report increased pupillary dilation in individuals with DS using tropicamide hydrochloride 0.01%, suggestive of differences in cholinergic function and more recently Nandakumar and Leat^7^ demonstrated increased pupillary dilation using cycloplentolate hydrochloride 0.5%. It is also widely reported that children with DS suffer from a range of systemic conditions affecting smooth muscle function including gastrointestinal tract problems^40^,^41^,^42^, reduced muscle tone and cardiac abnormalities^43^. The findings of the present study are compatible with an anatomical or innervational deficit of the sphincter pupillary muscle and ciliary muscle. To date the structure and function of the ciliary muscle in the DS eye has not been investigated nor has the thickness or shape of the crystalline lens been examined under accommodative demands.

Haugen *et al*.^5^ reported that the crystalline lens is thinner in participants with DS, and previous authors proposed that the mechanics of the DS lens may limit accommodative potential in a similar manner to presbyopia^2^,^4^,^6^,^8^,^10^, the normal impairment of accommodative function which occurs with age and which is attributed to the progressive reduction in flexibility of the ageing lens. Whilst *in-vivo* study of the DS lens during accommodation would be beneficial to explore lens structure further, it does not appear from the present data that children with DS are ‘early presbyopes’. Bharadwaj *et al*.^44^ report that presbyopes and incipient presbyopes demonstrate larger changes in pupil diameter in response to a near stimulus than those observed in children as a result of the increased neural effort required to generate a change in crystalline lens shape and hence an accommodative response in the ageing eye. If children with DS had a structural limitation of the crystalline lens, such as that which occurs through the natural aging process, a larger decrease in pupil diameter in response to an accommodative demand would have been expected in the present study. Conversely, the data suggest that anomalous innervation and/or musculature are implicated in hypoaccommodation in individuals with DS.

It may appear surprising that, given the increased prevalence of strabismus found in children with DS that the vergence response remains reliable despite the presence of hypoaccommodation. An abnormal relationship between the accommodative and vergence systems has been suggested as a possible cause of the accommodative deficit found in this population^3^,^5^,^6^. Haugen and Høvding^10^ suggest that esotropia in participants with DS may coincide with hypoaccommodation implemented in an attempt to control excessive convergence. Alternatively, Cregg *et al*.^4^ suggest convergence driven accommodation may be reduced or absent in children with DS. Subsequently, Stewart *et al*.^6^ demonstrated that children with DS who underaccommodated were more likely to be strabismic. In the present study, all three participants with DS and manifest strabismus during testing had hypoaccommodation. Further studies are required to clarify the interplay between accommodation and vergence and the role of accommodative-convergence and convergence-accommodation in children with DS.

## Conclusion

In conclusion, this is the first study to simultaneously and binocularly assess all three components of the near response in children with DS in comparison to typically developing controls. These data reveal that, despite their cognitive impairment, children with DS are attending and appropriately converging to near targets but frequently failing to accurately focus them. The data also demonstrate for the first time that hypoaccommodation is associated with acuity deficits. These findings improve our understanding of the near response in children with DS, indicating future research could fruitfully explore the possibility of a neurological or muscular anomaly of the accommodative apparatus in the DS eye and interrogate the relation between the visual components of the accommodative stimulus (retinal blur, disparity and proximity) and quality of focus.

## Additional Information

**How to cite this article**: Doyle, L. *et al*. Trying to see, failing to focus: near visual impairment in Down syndrome. *Sci. Rep.* **6**, 20444; doi: 10.1038/srep20444 (2016).

## Figures and Tables

**Table 1:** Table summarising the gender, mean age (±standard deviation (SD)), IPD (±SD) mean refractive error (±SD) and VA (±SD) of the better and worse seeing eye in participants with and without DS.

Group	Gender	Mean Age (Years ± SD)	IPD (mm ± SD)		Mean SER (D ± SD)	SER Range (D)	BCVA (logMAR ± SD)
DS (n = 24)	7 males	9.64 ± 2.96	53.20 ± 4.51	Better Seeing Eye	+2.18 ± 2.47	−2.25 to +7.88	0.24 ± 0.16
				Worse Seeing Eye	+2.20 ± 2.49	−2.00 to +7.63	0.34 ± 0.20
Controls (n = 75)	35 males	11.58 ± 3.13	56.82 ± 3.90	Better Seeing Eye	+0.65 ± 2.15	−6.00 to +8.25	−0.10 ± 0.07
				Worse Seeing Eye	+0.85 ± 2.18	−5.25 to +7.25	−0.03 ± 0.12

**Table 2:** Table summarising the mean (±SD), range and 5^th^ and 95^th^ centiles (mean response slope ± 1.96 × SD) of accommodative and vergence response slope values in both participants with and without DS.

Group	Accommodative Response Slope				Vergence Response Slope			
	Mean ± SD	Range	5^th^ & 95^th^ centile	P-Value	Mean ± SD	Range	5^th^ & 95^th^ centile	P-Value
DS (n = 24)	0.45 ± 0.32	0.04–1.19	0.08 to 1.15	<0.00001	0.90 ± 0.26	0.51–1.62	0.65 to 1.49	0.90
Controls (n = 75)	0.99 ± 0.25	0.45–1.70	0.61 to 1.52		0.90 ± 0.21	0.47–1.44	0.53 to 1.43	

**Table 3:** Table summarising the mean age (±SD), IPD (±SD) mean refractive error (±SD), BCVA (±SD) of the best and worse seeing eye and accommodative and vergence response slope value (±SD and range) in the REM control group (n = 34).

Mean Age (Years ± SD)	IPD (mm ± SD)		Mean SER (D)	SER Range (D)	BCVA (logMAR)	Mean Accommodative Response Slope	Mean Vergence Response Slope
12.31 ± 2.98	57.17 ± 3.64	Better Seeing Eye	+1.63 ± 2.59	−2.50 to +8.25	−0.09 ± 0.07	0.90 ± 0.25 (0.45 – 1.67)	0.94 ± 0.25 (0.61 – 1.44)
		Worse Seeing Eye	+1.89 ± 2.67	−2.50 to +7.25	−0.01 ± 0.10		
